# Nitric Oxide Prevents Alveolar Senescence and Emphysema in a Mouse Model

**DOI:** 10.1371/journal.pone.0116504

**Published:** 2015-03-10

**Authors:** Amanda E. Boe, Mesut Eren, Luisa Morales-Nebreda, Sheila B. Murphy, G. R. Scott Budinger, Gökhan M. Mutlu, Toshio Miyata, Douglas E. Vaughan

**Affiliations:** 1 Department of Medicine, Northwestern University Feinberg School of Medicine, Chicago, IL, United States of America; 2 Feinberg Cardiovascular Research Institute, Northwestern University Feinberg School of Medicine, Chicago, IL, United States of America; 3 Pulmonary and Critical Care Section, Department of Medicine, University of Chicago, Chicago, IL, United States of America; 4 United Centers for Advanced Research and Translational Medicine (ART), Tohoku University Graduate School of Medicine, Miyagi, Japan; University of Giessen Lung Center, GERMANY

## Abstract

N^ω^-nitro-L-arginine methyl ester (L-NAME) treatment induces arteriosclerosis and vascular senescence. Here, we report that the systemic inhibition of nitric oxide (NO) production by L-NAME causes pulmonary emphysema. L-NAME-treated lungs exhibited both the structural (alveolar tissue destruction) and functional (increased compliance and reduced elastance) characteristics of emphysema development. Furthermore, we found that L-NAME-induced emphysema could be attenuated through both genetic deficiency and pharmacological inhibition of plasminogen activator inhibitor-1 (PAI-1). Because PAI-1 is an important contributor to the development of senescence both *in vitro* and *in vivo*, we investigated whether L-NAME-induced senescence led to the observed emphysematous changes. We found that L-NAME treatment was associated with molecular and cellular evidence of premature senescence in mice, and that PAI-1 inhibition attenuated these increases. These findings indicate that NO serves to protect and defend lung tissue from physiological aging.

## Introduction

We have previously reported that chronic exposure to the nitric oxide synthase (NOS) inhibitor N^ω^-nitro-L-arginine methyl ester (L-NAME) causes hypertension, arteriosclerosis, and vascular senescence in mice [1,2,3]. The development of these pathologies can be attenuated through either genetic deficiency [1,2] or pharmacologic inhibition [3] of plasminogen activator inhibitor-1 (PAI-1). These studies defined the *in vivo* roles of both nitric oxide (NO) and PAI-1 in vascular senescence. While the vascular biology of NO often focuses on its vasodilator properties, NO can also alter proteins through posttranslational modification via S-nitrosylation to form S-nitrosothiols (SNOs). SNO-based signaling plays a major role in oxygen sensing, delivery, and utilization, and SNO-modified proteins affect the respiratory cycle, pulmonary gas exchange, and ventilation [4]. Therefore, we sought to expand upon our previous work and determine what role, if any, NO and PAI-1 has in lung tissue.

## Materials and Methods

### Experimental animals and L-NAME/TM5441 administration

Studies were performed on littermate 6–8 week old C57BL/6J mice (either WT or PAI-1^-/-^) of both sexes purchased from Jackson Laboratories (Bar Harbor, ME). L-NAME (Sigma Aldrich, St. Louis, MO) was administered in the drinking water at 1 mg/mL (approximately 100–120 mg/kg/day). TM5441 was mixed in the chow at a concentration of 20 mg/kg/day as described previously [3]. Mice remained in the study for either 1 week or 8 weeks before undergoing final measurements and tissue harvest.

### Ethics Statement

All work was performed as proposed in experimental Animal Study Protocol 2012–1771, which was approved by the Institutional Animal Care and Use Committee of Northwestern University. Euthanasia was carried out with Isoflurane followed by cervical dislocation for tissue harvest. Every effort was made to minimize suffering.

### qRT-PCR

Lungs harvested from mice were snap frozen in liquid nitrogen. 15–40 mg of tissue was weighed out for RNA isolation using the Qiagen RNeasy Mini Kit (Qiagen, Valencia, CA) by following the manufacturer’s protocol. cDNA was generated from the RNA using the qScript cDNA Supermix (Quanta Biosciences, Gaithersburg, MD) by following the manufacturer’s protocol. cDNA concentration was quantified using the Take 3 software and plate reader (BioTek Instruments, Winooski, VT). Samples were then diluted to generate 0.1μg/μL solutions.

Quantitative real-time PCR (qRT-PCR) was performed using the SsoAdvanced SYBR Green Supermix (Biorad, Hercules, CA) with primers for p16^Ink4a^ (F: 5’-AGGGCCGTGTGCATGACGTG-3’ and R: 5’-GCACCGGGCGGGAGAAGGTA-3’), p53 (F: 5’-GGCCCAAGTGAAGCCCTCCG-3’ and R: 5’-GCCCAGGGGTCTCGGTGACA-3’), p21 (F: 5’-GGACGTCCCACTTTGCCAGCAG-3’ and R: 5’-GAGCGCATCGCAATCACGGC-3’), and GAPDH (F: 5’-ATGTTCCAGTATGACTCCACTCACG-3’ and R: 5’-GAAGACACCAGTAGACTCCACGACA-3’) (Integrated DNA Technologies, Inc., Coralville, IA). Primers were resuspended at 100 μM and then diluted to generate 10 μM solutions. Reaction set up was as follows: 4.6 μL cDNA, 10 μL SYBR Supermix, 1 μL of both forward and reverse primers, 3.4 μL nuclease free water. Cycling conditions were: 95°C for 30 seconds, 40 cycles of 95°C for 5 seconds and 59°C for 15 seconds (CFX Connect Real-Time System, BioRad, Hercules, CA).

### Average telomere length ratio

Lungs harvested from mice were snap frozen in liquid nitrogen. Genomic DNA was isolated from 15–40 mg of lung tissue using the Qiagen DNeasy Blood & Tissue Kit (Qiagen, Valencia, CA) by following the manufacturer’s protocol. Telomere length was measured using quantitative real-time PCR as previously described with minor modification [5,6]. Briefly, telomere repeats are amplified using specially designed primers. These are then compared to the amplification of a single-copy gene, the 36B4 gene (acidic ribosomal phosphoprotein PO), to determine the average telomere length ratio (ATLR). Either 15 ng (aortas), 100 ng (livers), or 20 ng (lungs) of genomic DNA template was added to each 20 μl reaction containing forward and reverse primers (250 nM each for telomere primers, and 500 nM each for the 36B4 primers), SsoAdvanced SYBR Green Supermix (Biorad, Hercules, CA), and nuclease free water. A serially diluted standard curve of 25 ng to 1.5625 ng (aortas), 100 ng to 3.125 ng (livers), or 50 to 1.5625 (lungs) per well of template DNA from a WT mouse sample was included on each plate for both the telomere and the 36B4 reactions to facilitate ATLR calculation. Ct values were converted to ng values according to the standard curves. ng values of the telomere (T) reaction were divided by the ng values of the 36B4 (S) reaction to yield the ATLR. The primer sequences for the telomere portion were as follows: 5’-CGGTTTGTTTGGGTTTGGGTTTGGGTTTGGGTTTGGGTT-3’ and 5’-GGCTTGCCTTACCCTTACCCTTACCCTTACCCTTACCCT-3’. The primer sequences for the 36B4 single copy gene portion were as follows: 5’-ACTGGTCTAGGACCCGAGAAG-3’ and 5’-TCAATGGTGCCTCTGGAGATT-3’. Cycling conditions for both primer sets (run in the same plate) were: 95°C for 10 min, 30 cycles of 95°C for 15 s, and 55°C for 1 min for annealing and extension.

### FlexiVent

Mice were anesthetized, tracheostomized with a 20-gauge Angiocath, and placed on a forced system for measurement of lung mechanics with the use of a FlexiVent mouse ventilator (Scireq, Montreal, Canada). According to the protocols established by Scireq [7], a standard ventilation history for each mouse was first obtained with three total lung capacity (TLC) maneuvers to determine mean displaced volume (Vend), Vend relative to weight, and mean delivered volume. Then, a ‘‘snapshot perturbation” maneuver was imposed to measure resistance (R), compliance (C), and elastance (E). Subsequently, a forced oscillation perturbation (‘‘primewave-8”) was applied to measure airway resistance (Rn), inertia of the air, tissue damping (resistance) (G), elasticity (H), and tissue hysteresivity (tissue damping [G]/H). Finally, pressure-volume (PV) loops (PVs-V = PV-stepwise-volume regulated; PVr-V = PV-ramp-volume regulated; PVr-P = PV-ramp pressure regulated) were generated to obtain maximal vital (total) lung capacity (A), inspiratory capacity (IC) from zero pressure (B), form of deflating PV loop (K), static compliance (Cst), static elastance (Est), and hysteresis (area between inflating and deflating part of the loop).

For FlexiVent perturbations, a coefficient of determination of 0.95 was the lower limit for accepting a measurement. For each parameter, an average of three measurements was calculated per mouse.

### Lung inflation

A 20-gauge Angiocath was sutured into the trachea. The bronchi to the right lung was tied off so that the right lung could be removed for other analysis. The remaining left lung was inflated to 15 cm H_2_O with 10% paraformaldehyde (PFA) by attaching the Angiocath to a syringe filled with PFA that was elevated to 15 cm above the bench. This allowed gravity to force PFA into the lung equally in all samples, leading to expansion.

### Mean linear intercept calculation

The inflated lungs were paraffin embedded and sectioned at 6 microns. The lung was cut until clear, continuous branching of the bronchi was observed (slicing through the middle of the lung in the xy plane). Lung sections were stained with hematoxylin and eosin (H & E). The whole lung was then imaged at 20x objective using the TissueGnostics (Vienna, Austria) software and microscope located at the Northwestern University Cell Imaging Facility/Nikon Imaging Center (generously supported by NCI CCSG P30 CA060553 awarded to the Robert H. Lurie Comprehensive Cancer Center). The outline of the whole lung is traced, and the automated microscope takes serial 20x images covering the entirety of the traced area.

After imaging, 20 pictures from each lung were randomly selected using a random number generator to undergo mean linear intercept (MLI) quantification with the ImagePro software mentioned above. Each selected section had to be completely occupied by lung tissue and not contain large bronchi, vessels, or other miscellaneous tissue. A blank picture from that same slide was subtracted off of the selected 20 images for background correction. Using the color picking tools of the software, any area occupied by tissue was highlighted. Next, the overall image was converted into a binary picture and then inverted so that the tissue was colored black and the airspace colored white. A 6x7 grid was then superimposed on top of the black and white lung image. Using the operations tool, a new picture was generated showing where the grid and the lung tissue overlap. The number of intercepts was counted (including adding extra for any place where two of the grid lines intersected each other and tissue, as this would only be counted as one object by the software). MLI was determined by dividing the total length of the grid lines by the total number of intercepts (in this case, 11,541 μm/# intercepts).

### Statistical analysis

All results are presented as mean ± SD. Comparisons between 2 groups were tested by an unpaired, 2-tailed Student’s *t* test (unless otherwise noted). Results with P≤0.05 were considered significant.

## Results

**Fig 1:**
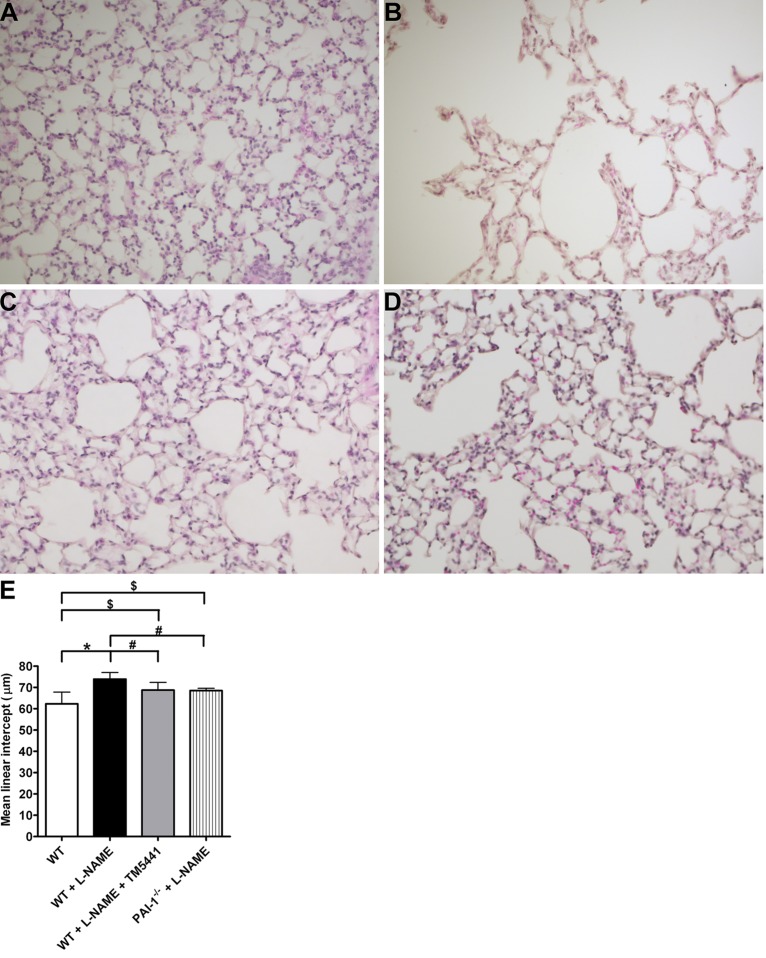
L-NAME treatment causes emphysema. Lung tissue sections from **(A)** WT, **(B)** WT + L-NAME, **(C)** WT + L-NAME + TM5441, and **(D)** PAI-1-/- + L-NAME demonstrate that L-NAME causes significant alveolar destruction and that PAI-1 inhibition is partially protective against this. **(E)** Mean linear intercept quantifications. *P = 0.0002, #P = 0.04, $P = 0.009. Data are mean ± SD. n = 11–13.

As with previous studies [1,2,3], we investigated the effects of L-NAME (1 mg/mL) or regular water for 8 weeks in 6–8 week old C57BL/6J mice [8]. We observed that L-NAME-treated mice exhibited a substantial amount of alveolar tissue obliteration resembling emphysema (Fig. 1B) compared to controls that ingested unmodified drinking water (Fig. 1A). Since loss of PAI-1 activity has been shown to be protective against L-NAME-induced pathologies, we evaluated whether genetic deficiency or pharmacologic inhibition of PAI-1 would attenuate L-NAME-induced emphysema. This was accomplished by either administering L-NAME to PAI-1 knockout (PAI-1^-/-^) mice or by co-treating WT mice with L-NAME and the small molecule antagonist TM5441 (described previously in [3]). Compared to animals on L-NAME alone, the L-NAME + TM5441 and the PAI-1^-/-^ + L-NAME mice had substantially less alveolar tissue loss (Fig. 1C and 1D). We quantified the extent of emphysema in these animals by calculating mean linear intercept (MLI) in lung histology sections. As shown in Fig. 1E, animals given L-NAME had a higher MLI compared to WT (72.7 ± 4.0 μm vs. 62.3 ± 5.5 μm, P = 0.0002). However, this increase was partially attenuated by both genetic deficiency (68.5 ± 3.4 μm, P = 0.04 vs. WT + L-NAME) and pharmacologic inhibition (68.7 ± 3.6 μm, P = 0.04 vs. WT + L-NAME) of PAI-1.

**Fig 2:**
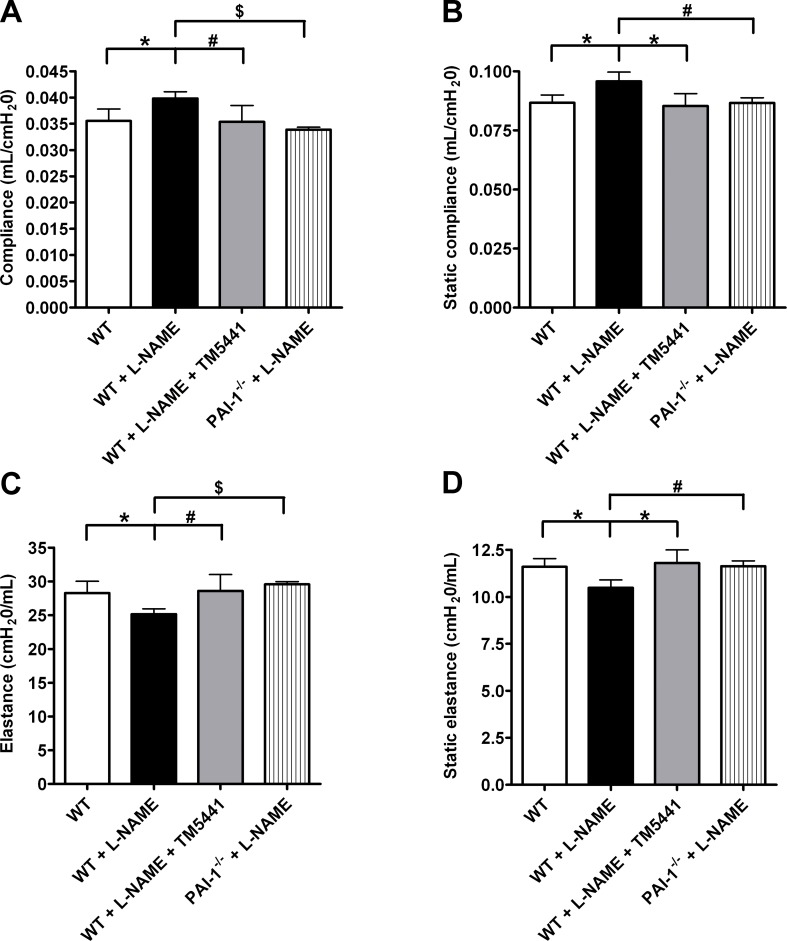
Effects of L-NAME on lung functional dynamics. Measurements for **(A)** compliance, **(B)** static compliance, **(C)** elastance, and **(D)** static elastance demonstrate that L-NAME-treated lungs have the functional characteristics of emphysema. Both genetic and pharmacologic inhibition of PAI-1 protected mice from lung dysfunction. **(A)** and **(C)** *P = 0.002, #P = 0.02, $P = 6.3x10^-6^. **(B)** and **(D)** *P = 0.004, #P = 0.01. Data are mean ± SD. n = 6–7.

We also measured lung functional dynamics using the FlexiVent mouse ventilator. Compliance and elastance were each measured 2 ways using different perturbations. As shown in Fig. 2, we found that animals treated with L-NAME had both a higher compliance (C) and static compliance (Cst) than WT animals (P = 0.002 and P = 0.004, respectively), consistent with the development of emphysema. The same was true for the inverse measurement, as L-NAME-treated animals had lower values for both elastance (E) and static elastance (Est). However, when L-NAME was administered to either PAI-1-deficient animals or mice treated with TM5441, the values for C, Cst, E, and Est were all similar to WT controls, indicating that these animals were protected against emphysema development. These FlexiVent findings are consistent with the interpretation that PAI-1contributes to the development of L-NAME-induced emphysema.

**Fig 3:**
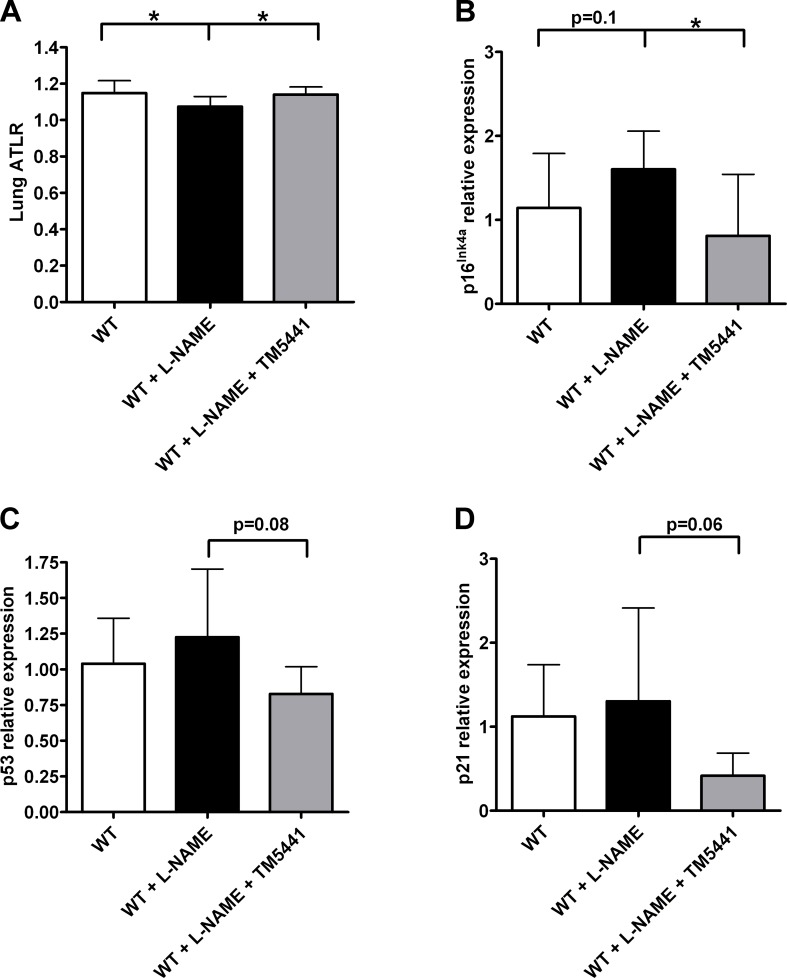
L-NAME-induced senescence in lung tissue. **(A)** ATLR measurements from 8 week-treated lungs. **(B-D)** qRT-PCR data from 1 week-treated lungs evaluating the senescence markers **(B)** p16^Ink4a^, **(C)** p53, and **(D)** p21. **(A)** *P = 0.007. n = 12. **(B-D)** *P = 0.02. n = 7–11. Data are mean ± SD.

Since there is no precedent in the literature of using chronic L-NAME administration as a model for emphysema, we investigated the mechanism behind alveolar tissue loss in these animals. First, we examined if L-NAME could promote the development of senescence similar to that previously seen in the aorta [3]. After 8 weeks of treatment, telomere length in the lung of L-NAME-treated animals was decreased (P = 0.007 vs. WT), and TM5441 co-treatment prevented this reduction (P = 0.007 vs. WT + L-NAME) (Fig. 3A). However, when the expression levels of the senescence marker p16^Ink4a^ were measured, there was no difference between L-NAME-treated and untreated mice. Since emphysema is already established at 8 weeks, we hypothesized that the cells that had expressed p16^Ink4a^ may have been already cleared, leading to the tissue loss characteristic of emphysema. Therefore, we looked at earlier time points in order to identify evidence of senescence prior to the development of emphysema. As shown in Fig. 3B, after one week on L-NAME, p16^Ink4a^ expression was increased in the lung. Notably, there was a detectable difference in p16^Ink4a^ expression between the L-NAME and the L-NAME + TM5441 groups at this early time point (P = 0.02). Although not significant, a similar pattern is also seen when looking at other senescent markers such as p53 (Fig. 3C) and p21 (Fig. 3D). This data indicates that L-NAME triggers cellular senescence relatively rapidly after initiating the treatment. Together with the telomere length results, these findings indicate that L-NAME-induced emphysema may be mediated through cellular senescence.

## Discussion

The present study represents the first report of using L-NAME treatment to induce emphysema. However, this work is not the first to examine the role of NOS inhibition in lung disease. Several prior reports have focused on the role of iNOS in emphysema. In general, these studies indicate that inhibition of iNOS, either pharmacologically or through genetic knockout, protects against the development of emphysema [9,10]. Additionally, a single study found that in a cigarette smoke (CS)-induced model, both L-arginine and L-NAME were somewhat protective against the development of emphysema [11].

In general, the existing literature seems to contradict our findings, as the reduction of NO production is associated with protection against emphysema. However, these results can be explained by examining the differences in the respective models used. In each of these previous studies, the authors used another stimulus (CS, elastase) to trigger emphysema initially before investigating the isolated and selective effects of NO. Cigarette smoke and elastase both trigger an inflammatory response that is associated with intense augmentation of iNOS expression. When stimulated, iNOS produces a large amount of NO. Furthermore, iNOS activation usually occurs in an oxidative environment, which allows for NO to react with superoxide to form peroxynitrite. Normally, this functions as part of the immune response and aids in the destruction of bacterial or tumor cells. However, in both the CS and elastase models of emphysema, excess NO production from iNOS led to further tissue destruction through oxidative stress, which was associated with increased levels of peroxynitrite-induced apoptosis and reduced proliferation of alveolar epithelial cells, a key step in the development of emphysema [10]. This likely explains why previous reports found that iNOS inhibition was protective against emphysema in the CS and elastase models. Since L-NAME inhibits all three forms of NOS, the previous finding that L-NAME is protective against emphysema is likely due a reduction in iNOS-derived NO.

The administration of L-NAME alone does not appear to cause an inflammatory response, and therefore in this model iNOS is likely not activated. Instead, emphysema in this model appears to be the result of vascular senescence triggered by a lack of NO production from eNOS [12]. While generating emphysema by chronic L-NAME administration is a novel approach, other studies have demonstrated a pivotal role of endothelial cell function in lung disease. Both angiogenesis and vascular remodeling (two processes which rely on NO production) have been hypothesized to play critical roles in emphysema [13]. In guinea pigs, CS leads to reduced lung expression of eNOS and endothelial dysfunction of the pulmonary arteries, both of which preceded the development of emphysema [14]. Interestingly, in this same CS guinea pig model, co-treatment with a statin increased NO production and protected against emphysema [15]. Furthermore, eNOS has been shown to be important for compensatory lung growth. Both eNOS knockout mice and L-NAME-treated animals have impaired growth in a pneumonectomy model due to a lack of alveolar cell proliferation and reduced angiogenesis [16]. Alveolar repair in elastase-treated rats was also found to be positively correlated with eNOS expression by vascular regeneration [17]. Finally, vascular endothelial growth factor (VEGF), a known inducer of NO production, has also been implicated in the pathogenesis of emphysema [18]. Both lung-specific VEGF knockout mice [19] and mice treated with a VEGF receptor inhibitor [20] developed emphysema.

In humans, smokers have been shown to have reduced eNOS expression in their pulmonary arteries compared to non-smokers [21], along with decreases in both VEGF and VEGFR expression [22]. Smokers with emphysema had shorter telomeres and increased expression of p16 in both endothelial cells and alveolar type II cells [23]. Additionally, NO plays a major role in oxygen sensing, delivery, and utilization through SNO-based signaling. SNO-modified proteins affect the respiratory cycle, pulmonary gas exchange, and ventilation [4].

Our data expands upon this previous work, but is the first to directly look at the role of L-NAME in emphysema. We examined telomere length and p16^Ink4a^ levels in the lung tissue to determine if the emphysematic pathology was a consequence of L-NAME-induced senescence. This hypothesis was confirmed, as L-NAME treatment resulted in significantly shorter telomeres after 8 weeks and an increase in p16^Ink4a^ expression after just one week. Genetic deficiency and pharmacological inhibition of PAI-1 protected against the development of L-NAME-induced emphysema, as shown by both functional and histological assessments of the lungs. Furthermore, PAI-1 antagonism both preserved lung telomere length and attenuated the increases in p16^Ink4a^, p53, and p21 expression. These results further support the idea that PAI-1 is a critical determinant of vascular senescence, and that L-NAME-induced emphysema is one of several pathological consequences of this senescence. Interestingly, previous work has shown that PAI-1 is upregulated in the sputum and macrophages of patients with chronic obstructive pulmonary disorder (COPD), and that it could play a pro-inflammatory role in the pathogenesis of emphysema [24,25]. Furthermore, this increase in expression was due to oxidative stress [26]. Thus, in addition to its pro-senescent role, elevated PAI-1 may contribute to emphysema through other molecular pathways. This would further explain the protection against tissue destruction seen with genetic deficiency or TM5441-mediated inhibition of PAI-1.

While more work is needed to define fully the mechanism behind L-NAME-induced emphysema, the present findings demonstrate that the interplay between NO, PAI-1, and vascular senescence has an important impact on alveolar tissue integrity. L-NAME treatment represents a novel model for emphysema pathogenesis that could be useful in future studies.
